# Host identity and phylogeny shape the foliar endophytic fungal assemblages of *Ficus*

**DOI:** 10.1002/ece3.5568

**Published:** 2019-08-13

**Authors:** Junwei Liu, Jin Zhao, Gang Wang, Jin Chen

**Affiliations:** ^1^ CAS Key Laboratory of Tropical Forest Ecology Xishuangbanna Tropical Botanical Garden Chinese Academy of Sciences Mengla Yunnan China; ^2^ University of Chinese Academy of Sciences Beijing China

**Keywords:** *Ficus*, foliar endophytic fungi, leaf functional traits, phylogenetic signal

## Abstract

Foliar endophytic fungi (FEF) are diverse and ubiquitously associated with photosynthetic land plants. However, processes shaping FEF assemblages remain poorly understood. Previous studies have indicated that host identity and host habitat are contributing factors, but these factors are often difficult to disentangle. In this study, we studied FEF assemblages from plants grown in a botanical garden, enabling us to minimize the variation in abiotic environmental conditions and fungal dispersal capacity. FEF assemblages from 46 *Ficus* species were sequenced using next‐generation methods, and the results indicated that closely related host species had clearly differentiated FEF assemblages. Furthermore, host phylogenetic proximity was significantly correlated with the similarity of their FEF assemblages. In the canonical correspondence analysis, eleven leaf traits explained 32.9% of the total variation in FEF assemblages, whereas six traits (specific leaf area, leaf N content, leaf pH, toughness, latex alkaloid content, and latex volume per leaf area) were significant in the first two dimensions of ordination space. In the multiple regression on distance matrix analysis, 21.0% of the total variance in FEF assemblage was explained by both host phylogeny and leaf traits while phylogeny alone explained 7.9% of the variance. Thus, our findings suggest that both evolutionary and ecological processes are involved in shaping FEF assemblages.

## Graphical abstract

Host plant plays a key role in shaping the foliar endophytic fungi assemblage, host phylogenetic proximity was significantly correlated with the similarity of their FEF assemblages, and 21.0% of the total variance in FEF assemblage was explained by both host phylogeny and leaf traits while phylogeny alone explained 7.9% of the variance.

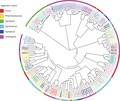

## 1 INTRODUCTION

Endophytic fungi live within plant tissues without causing visible symptoms (Petrini, 1991; Wilson, 1995). They have been associated with plants for over 400 Myr (Krings et al., 2007) and have been found in all tested plant species (Arnold, 2007), including trees (Arnold, Maynard, Gilbert, Coley, & Kursar, 2000; Carroll, Muller, & Sutton, 1977), palms (Frohlich, Hyde, & Petrini, 2000), grasses (Clay, 1988), mistletoes (Persoh, 2013), lycophytes (Higgins, Arnold, Miadlikowska, Sarvate, & Lutzoni, 2007), and nonvascular plants (Davis & Shaw, 2008; Desiro, Duckett, Pressel, Villarreal, & Bidartondo, 2013). Foliar endophytic fungi (FEF) form distinct fungal guild (Arnold, 2007), although sharing species with others, and are highly diverse even within a single plant, ranging from 10 to >200 species per tree (Arnold, 2007; Hyde & Soytong, 2008; Zimmerman & Vitousek, 2012). FEF are of particular interest to ecologists owing to their potential capacity to modify the fitness of a host plant, for example, by providing protection from microbial pathogens (Arnold et al., 2003) or by reducing herbivore fecundity (González‐Teuber, 2016; Van Bael et al., 2009). As numerous FEF live in an individual plant constituting an assemblage, understanding how these assemblages are shaped is a fundamental mission of ecologists and may very likely improve our ability to manipulate assemblages for human welfare (Zambell & White, 2017).

Multiple factors play a role in determining the composition of FEF assemblages, such as the dispersal capacity of fungi, abiotic habitat filtering, host‐imposed habitat filtering, and interactions among fungal species (Saunders, Glenn, & Kohn, 2010). Environmental factors have been well documented to influence FEF assemblages (Eschen, Hunt, Mykura, Gange, & Sutton, 2010; Whitaker, Reynolds, & Clay, 2018; Zimmerman & Vitousek, 2012). Pan and May (2009) found that interspecific interactions among fungi can affect endophytic assemblages. Schulz, Boyle, Draeger, Rommert, and Krohn (2002) found that approximately 80% of fungal endophyte species produce secondary compounds with antifungal activity, which mediated the competition among fungal species (Saunders et al., 2010). Previous studies have also indicated that different host species show distinct FEF assemblages (Higgins et al., 2007; Solis, Dela Cruz, Schnittler, & Unterseher, 2016; Vincent, Weiblen, & May, 2016). An early study of FEF assemblages on coniferous trees found that many endophytes were restricted to a single or a restricted number of host species (Carroll & Carroll, 1978). For co‐occurring host species, the host species was also found to have a significant effect on endophytic fungal assemblages (Persoh, 2013). Even closely related host species were found to have distinct FEF assemblages, as shown in a study on three *Nicotiana* species and their endophytes (Dastogeer, Li, Sivasithamparam, Jones, & Wylie, 2018). Another study indicated that fungal assemblages of conspecific host individuals were significantly more similar than those of heterospecific hosts (U'Ren, Lutzoni, Miadlikowska, Laetsch, & Arnold, 2012).

However, several questions relevant to the influence of host‐imposed habitat filtering on its FEF assemblage are still unanswered. Firstly, whether host identity is a key factor shaping FEF assemblages require more evidence. On one hand, a study on tropical grasses found that most nonsingleton FEF isolates occurred in more than one genus of Poaceae, suggesting broad host generalism of fungal endophytes (Higgins, Coley, Kursar, & Arnold, 2011). On the other hand, previous studies manifesting host's influence include either phylogenetically distant species (Vincent et al., 2016) or only a limited number of congeneric host species. Studies examining a larger range of closely related host species could strengthen our understanding of the determinant influence of host identity. Secondly, whether host phylogeny correlates to similarities among FEF assemblages is still unknown. Phylogenetic conservatism influences associations in the coevolved interactions between plants and pathogenic fungi and plants and mycorrhizal fungi (Gilbert & Webb, 2007; Shefferson et al., 2010). However, Vincent et al. (2016) attempted to detect the influence of host phylogenetic distance on the dissimilarity of FEF among 11 plant species from five genera (*Ficus*, Moraceae; *Macaranga*, Euphorbiaceae; *Psychotria*, Rubiaceae; *Syzygium*, Myrtaceae; and* Gnetum*, Gnetaceae) and found no significant influence. Thirdly, which leaf traits contribute more to FEF assemblage is still unclear. Leaf traits are key factors in host filtering for foliar endophytic fungi. Arnold et al. (2003) found that FEF grew faster on leaf extracts of species they abundantly colonized than those they did not, suggesting that host‐specific leaf traits mediate interactions within endophytes, thereby influencing endophyte species composition. In another study, Vincent et al. (2016) found that three leaf traits explained up to 22% of the variation in FEF assemblages. The inclusion of a larger number of leaf traits could increase our knowledge of factors affecting FEF assemblages.

Plant–FEF interactions occur as part of complex ecological networks (Rai & Agarkar, 2016; Rodriguez, White, Arnold, & Redman, 2009); however, it is difficult to decouple the influences of dispersal capacity, abiotic environmental filtering, and host‐imposed habitat filtering. Studying FEF assemblages in a botanical garden, in which multiple host species live in similar environmental conditions and share the same fungal meta‐community in a relatively small site, may limit the variations due to differences in fungal dispersal capacity and in abiotic environmental conditions, providing an opportunity to better understand other contributing factors, such as host‐imposed habitat filtering.

*Ficus*, pantropical in distribution, is one of the largest woody genera in the tropics, with about 750 species and a wide range of life forms (Berg, 1989; Stevens, 2001). Following Berg (2003), *Ficus* is currently classified into six subgenera, that is, *Pharmacosycea*, *Urostigma*, *Sycomorus*, *Ficus*, *Sycidium*, and *Synoecia*. *Ficus* have been recognized as a keystone species in tropical rainforests because they provide a year‐round food source for many frugivorous animals (Sanitjan & Chen, 2009; Shanahan, So, Compton, & Corlett, 2001). Two studies on the FEF of *Ficus* (Solis et al., 2016; Vincent et al., 2016) have suggested that host identity affects FEF assemblages. In this study, we used the fig collection in the Xishuangbanna Tropical Botanical Garden (XTBG), in which all fig trees grow in a small area (1.3 ha), in the same seminatural environment, in a humid tropical climate, and are exposed to the same community of herbivores and pathogens, to address the following questions: (a) does host identity influence FEF assemblages; (b) does phylogeny of host plants significantly affect FEF assemblages; and (c) do leaf traits filter FEF and contribute to explain the variation in FEF assemblages.

## 2 MATERIAL AND METHODS

### 2.1 Conditions in Xishuangbanna Tropical Botanical Garden

The fig collection in Xishuangbanna Tropical Botanical Garden (XTBG; 21°56′N, 101°15′E, 600 m a.s.l.) contains about 80 species and varieties of the genus in an area of 1.3 ha, including all of the six subgenera, multiple life forms, and two sexual systems (monoecious and dioecious). The collection is managed without the use of pesticides or fungicides; thus, all plants are exposed to natural fungi and herbivorous insects. In addition, most of plants have historic records, including provenance and propagation history, most of the species used in this study are native to the Yunnan province, and all the species were introduced into the garden before 2011 (Table S1).

### 2.2 Sample collection

Leaves were only sampled from well‐developed *Ficus* individual during the wet season in 2015, when most fig species produce new leaves. Ten well‐expanded mature and healthy leaves were sampled from each individual (Unterseher, Reiher, Finstermeier, Otto, & Morawetz, 2007). Only six leaves were sampled from *F. tikoua* due to limitations in the leaf availability. Leaves from each *Ficus* individual were pooled to form one sample. Totally, 166 samples from 46 species were collected to investigate FEF assemblages and individual number sampled and sequenced for each studied species was presented in Table S1. The number of individuals of each *Ficus* species ranged from one to greater than ten. For species with only one or two individuals, we did not measure leaf traits. For several species with three or more individuals, we were not able to sample enough leaves both for measuring leaf traits and surveying FEF assemblages owing to the limited availability of leaves meeting our criteria. A further limitation in collecting the leaves was due to the living collections management policy of XTBG, which only allowed us to collect some of the new leaves. For those species with more than three individuals available, three individuals were opportunistically selected. Totally, 69 samples from 23 species were collected for measurement of leaf traits (Table S2).

### 2.3 Measurement of leaf traits

Eleven leaf traits were measured, including specific leaf area (SLA, cm^2^/g dry mass), leaf water content (%), total C content (g/kg), N content (g/kg), pH, toughness (g), leaf tannin content per mass (%), latex volume per area (μl/cm^2^), latex water content (%), latex tannin content (g/L), and latex alkaloid content (g/L) (Table S2). Leaf traits were selected based on the following considerations: leaf N content has relevant functional traits in leaf economics (Wright et al., 2004); plant investment in latex can assist with sealing wounds, deterring herbivory (Bauer et al., 2014), and possibly storing nutrients and water (Hunter, 1994); secondary metabolites inhibiting growth of fungi have been isolated from the latex (Upadhyay, 2011); leaf pH shows significant interspecific variation (Cornelissen, Sibma, Van Logtestijn, Broekman, & Thompson, 2011); and the proliferation of single fungal species and communities was influenced by pH (Mehra & Jaitly, 1995; Rousk, Brookes, & Bååth, 2009). Other leaf traits are frequently measured in ecological research but their potential role in influencing FEF assemblages has not been studied.

Leaf area (cm^2^) was measured with digital photographs using ImageJ (Version 1.46r). Leaf material and latex were oven‐dried at 40°C until a constant mass was reached. SLA was estimated as the ratio of leaf area to leaf dry mass. Water content was estimated as the difference between the wet and dry mass for both leaf and latex. Dry samples were then sent to the XTBG Central Laboratory for measuring chemical properties. Total C and N were determined by Dumas combustion analysis (Dumas, 1831) using an elemental analyzer (Vario MAX CN; Elementar). The pH was measured following Cornelissen et al. (2011). Each fresh subsample was manually chopped into pieces of ~1 mm and mixed with demineralized water in a 2.5 ml Eppendorf tube (volume ratio 1:8). After 1 hr of shaking at 250 rpm, the solution was centrifuged at 12,000 *g* and the supernatant was measured using a PHS‐3C pH meter (LeiCi). The pH meter was calibrated against buffer solutions (pH 4, 7, and 9) before each measurement series. Toughness was measured with a device modeled after a design by Feeny (1970) and presented as the weight required punching the leaves.

Latex exudation was collected by cutting the leaf tips. The latex volume was measured by a silanized glass capillary and then placed in a preweighed Eppendorf tube. These tubes were weighed by an electronic balance in the XTBG Central Laboratory. Then, the latex‐containing Eppendorf tubes were oven‐dried at 45°C for 72 hr to measure the dry amount, thus estimating the water content of the latex. A further 10 μl of latex (from the cut petiole after the leaf was removed) was collected from the leaves, dissolved in 90 μl of purified water, and diluted 10 times. The mixture was filtered through a 0.45‐µm membrane. The extract was fully blended with 50 ml acetone for 40 min and filtered through a medium speed quantitative filter. Then, 1 ml of filtrate was mixed with 2.5 ml of sodium tungstate–phosphorus‐molybdenum acid mixed solution and 5 ml of carbonate. The tannin content was measured by a colorimetric method using the Folin–Denis reagent (Bajaj & Devsharma, 1977). The bromothymol blue colorimetric method was used to determine the latex alkaloid content (Shamsa, Monsef, Ghamooshi, & Verdianrizi, 2008).

### 2.4 Sample preparation, DNA extraction, PCR, and sequencing

In our study, only endophytic fungi were taken into consideration; fungi in the phylloplane were excluded using a rigorous sterilization procedure. The surfaces of all leaves were sterilized within 24 hr to reduce the presence of phyllosphere microorganisms. The leaves were rinsed in deionized (di) H_2_O, immersed in 95% (vol/vol) ethanol (5 s), 0.5% NaClO (2 min), and 70% vol/vol ethanol (2 min), and finally rinsed three times for 1 min in di H_2_O (Arnold, Henk, Eells, Lutzoni, & Vilgalys, 2007). This treatment proved effective in removing any microbes present on the leaf surface (Arnold et al., 2007; Zimmerman & Vitousek, 2012). The sterilized leaves were then dried in biosafety cabinets and stored at −80°C after rapid cooling in liquid nitrogen. After all the samples were collected, the leaves from the same individual host were pooled and ground together in a mortar to produce a single sample.

Total DNA was extracted from the subsamples of pooled leaves using the HP Plant DNA Mini Kit (OMEGA Bio‐tek) and stored at −20°C. Then, the ITS1 region (Nilsson et al., 2019) was PCR‐amplified on a BioRad thermal cycler (BioRad) in triplicate to reduce the effects of amplification bias. Reactions were carried out in a 20 μl volume with 10 μl of 2× Power Taq PCR Master Mix (BioTeke), 1 μl of DNA template, 0.5 μl each of 20 μM forward and reverse primers, and 8 μl of sterilized water (purified using a Milli‐Q A10; Merck, Millipore). The amplification system was prepared on ice to reduce the formation of primer dimers. To allow for the postsequencing differentiation of the samples, 16 pairs of distinct forward and reverse primers were customized by adding a distinct short barcode to the 5′ end of the fungal‐specific primer ITS1‐F (5′‐CTTGGTCATTTAGAGGAAGTAA‐3′) and reverse primer ITS2 (5′‐GCTGCGTTCTTCATCGATGC‐3′) (Schoch et al., 2012).

The hot‐start PCR reactions were cycled for 3 min at 94°C, followed by 30 cycles of 1 min at 94°C, 30 s at 54°C, 1 min at 72°C, and, finally, 7 min at 72°C. Negative controls were run for DNA extractions (100 ml of sterilized Milli‐Q water substituted for plant material) and PCR (1 μl sterilized Milli‐Q water substituted for the DNA template); no amplicons were detected by electrophoresis. Each triplicate reaction was pooled, and PCR cleanup was performed using Bioteke purification kits (BioTeke) according to the manufacturer's instructions. After testing the cleaned PCR products for concentration using a DNA assay 2100, and for size via gel electrophoresis, amplicon samples were standardized by molarity and pooled into a single sample. Libraries were built using a combination of PKR Truseq Adapter Kit (Beijing Pukairui Biological Technology Co. Ltd.) and KAPA Hyper Prep Kit (Roche) and then quantified by quantitative real‐time PCR (QPCR) according to the KAPA Library Quantification Kit's protocol (Roche). The PKR Truseq Adapter Kit provides 12 unique adapters, each containing one unique index, which are compatible with Illumina's MiSeq platform and allow demultiplexing the data postsequencing by the platform software. The libraries were pooled following Illumina's guidelines and sequenced on Illumina's MiSeq platform, using the MiSeq Reagent Kit V2 (Illumina) which allows a maximum read length of 2 × 250 bp by paired‐end sequencing. In this study, we did not conduct negative controls for the sequencing, due to our pilot study indicated a very limited influence of extremely rare sequences in downstream analysis, compared with abundant sequences in samples with much higher concentration of amplicons.

### 2.5 Bioinformatics

The raw data from MiSeq were primarily grouped according to the unique index of each library by the platform software. Each library produced two files containing Read1 and Read2 sequences in FASTQ format. Paired‐end reads were merged using USEARCH 10.0 (Edgar, 2010). The merged sequences were demultiplexed via the unique barcode present in both forward and reverse primer using fastx_toolkit 0.0.13.2 with zero mismatches (http://hannonlab.cshl.edu/fastx_toolkit/). Primers and barcodes were trimmed using USEARCH 10.0 and served as raw sequences to produce the operational taxonomic units (OTU) table. Amplicon sequence data were deposited in a Sequence Read Archive under accession number SRR8675934 (National Center for Biotechnology Information, US) and Dryad Digital Repository. To obtain high‐quality sequences for clustering OTUs, the reads were further filtered by USEARCH 10.0 with a constant maximum expected error threshold of one. Sequences with homopolymers with length >10 bp or with a total length of <100 bp were discarded according to variance of ITS1 length (Toju, Tanabe, Yamamoto, & Sato, 2012) using Mothur (Schloss et al., 2009). Then, the sequences from all samples were pooled and dereplicated by VSEARCH 2.4.4 (Rognes, Flouri, Nichols, Quince, & Mahé, 2016) to obtain unique sequences. A similarity threshold of 97% was chosen (Bálint, Schmidt, Sharma, Thines, & Schmitt, 2014; Lindahl et al., 2013) to cluster OTUs using USEARCH 10.0. The OTU table was built using VSEARCH 2.4.4, and singletons in each sample were removed before downstream analysis.

### 2.6 Phylogeny of *Ficus*

Three gene loci were downloaded from the NCBI for each of the 46 *Ficus* species, and sequence accession numbers are given in the supporting information (Table S3). For each locus, alignment and end‐trimming were separately conducted using MEGA 6 (Tamura, Stecher, Peterson, Filipski, & Kumar, 2013) and linked by Geneious 11.0.2 (Kearse et al., 2012). Nucleotide substitution models of the linked sequences were tested by jModelTest 2.1.10 (Posada, 2008), and the model of GTR + I+G was chosen according to Akaike information criterion (Akaike, 1974). A Bayesian tree was created, based on the linked sequences, through a combination of SequenceMatrix‐Windows‐1.8 (Vaidya, Lohman, & Meier, 2011) and BEAST.v2.4.5.Windows (Bouckaert et al., 2014). *Antiaropsis decipiens* and *Castilla elastica* were used as outgroups. The phylogeny and tree file in Newick format are given in the Table S3, Figure S1, and Tree S1. The phylogenetic tree was pruned to remove outgroups in testing the relationship between host phylogeny and similarity among FEF assemblages. It was further pruned to fit the species number in detecting phylogenetic signals of leaf traits and the influence of leaf traits and host phylogeny on FEF assemblages, owing to data availability, using the “drop.tip” function in the ape package of R (Paradis & Schliep, 2018).

### 2.7 Statistical analyses

All statistical analyses and data manipulations were carried out using R 3.4.3 (R Core Team, 2017). All functions were implemented using the vegan package (Oksanen et al., 2013), unless explicitly stated. The OTU tables were transformed to proportional abundance of OTUs to total reads counts in a given sample using the “decostand” function. Bray–Curtis dissimilarity was used to evaluate the distance between FEF assemblages. To evaluate whether sequencing depth captured the composition of FEF assemblages, OTU rarefaction curves were obtained using the “rarecurve” function.

To detect the impact of closely related host species on FEF assemblages, three subsets were firstly extracted from the OTU table according to species composition. Here, “closely related species” refers to species that were consistently located in the same subsections in the phylogenetic tree or within *Ficus auriculata* species complex. The differences in FEF assemblages among closely related species were evaluated using analysis of similarities (ANOSIM) and permutational multivariate analysis of variance (PERMANOVA) (Anderson, 2001), implemented with the “anosim” and “adonis” functions in vegan package. Given the potential susceptibility of PERMANOVA to elevated type I error in the case of heterogeneous variance among groups, homogeneity of variance was examined using global and pairwise comparisons with a permutation test (999 permutations), implemented with the “betadisper” function (bias.adjust = TRUE). Detrended correspondence analysis (DCA) was used to visualize the similarities among closely related host species. To alternatively visualize the impact of host identity, hierarchical clustering was used to group the FEF assemblages from each *Ficus* individual with the function “hclust” in the stats package.

The Mantel test was used to test the relationship between the dissimilarities in FEF assemblages and the phylogenetic distance among host species, using the “mantel” function in the ecodist package (Goslee & Urban, 2007). The phylogenetic tree was pruned to include only the 46 *Ficus* species studied (Table S1). OTUs of all individuals for a given *Ficus* species were pooled to form one sample by summing the reads counts before the Mantel test, which referred to the FEF assemblage of the species. The influence of subgenus was also evaluated with the “anosim” function owing to the variation in the number of individuals. Principal coordinate analysis (PCoA) was used to visualize the dissimilarity among subgenera, using the “capscale” function.

Due to the variation of individual number of *Ficus* and material availability when sampling, the impact of the 11 measured leaf traits on FEF assemblages was only tested for 23 *Ficus* species that were represented by three individuals each (Table S2). The subset was extracted from the OTU table according to the species list. Canonical correspondence analysis (CCA) was used to test the combined impacts of leaf traits on FEF assemblages, and the “envfit” function was used to fit leaf traits onto a CCA ordination. The phylogenetic signals of leaf traits were detected using the *K* statistic (Blomberg, Garland, & Ives, 2003) to compare the observed trait distribution in the community of samples with the expected under the Brownian motion model of trait evolution. Calculations of *K* values were performed in the picante package (Kembel et al., 2010). The simple Mantel test and partial Mantel test were used to detect the influence of each leaf trait on FEF assemblages with the impact of host phylogeny contained and controlled, respectively, using the “mantel” function in the ecodist package. The significance and relative contribution of leaf traits were also tested by multiple regression on distance matrix analysis, using the function “MRM” in the ecodist package. The fraction of the variation explained by each predictor variable in the multiple regression was calculated according to Krasnov, Mouillot, Shenbrot, Khokhlova, and Poulin (2010) and presented as a percentage.

## 3 RESULTS

After global trimming and quality control, a total of 6,557,673 high‐quality sequences (mean length, 307 bp) from 8,096,909 raw sequences were used to construct the OTU list, and 5,353 OTUs were grouped at 97% similarity. All the raw sequences were used to build the OTU table. The sequence‐based rarefaction curves based on the observed OTUs were asymptotic (Figure S2), indicating that sequencing depth was adequate for capturing the composition of FEF assemblages at the individual level.

### 3.1 Influence of host identity in shaping FEF assemblages

**Figure 1:**
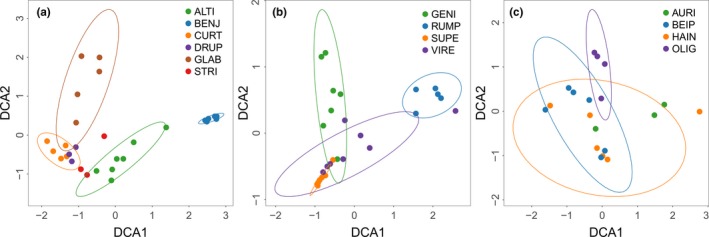
Two‐dimensional DCA ordination showing the influence of host species on the structure of FEF assemblages in phylogenetically closely related species (a: subsection *Conosycea*; b: subsection *Urostigma*; c: *F. auriculata* species complex). Abbreviations are the first four letters of specific epithets of host species (Table S1). Ellipses indicate the location and dispersion in ordination space for each host species represented by more than three individuals (confidence level = 0.90). Points represent host individuals

**Figure 2:**
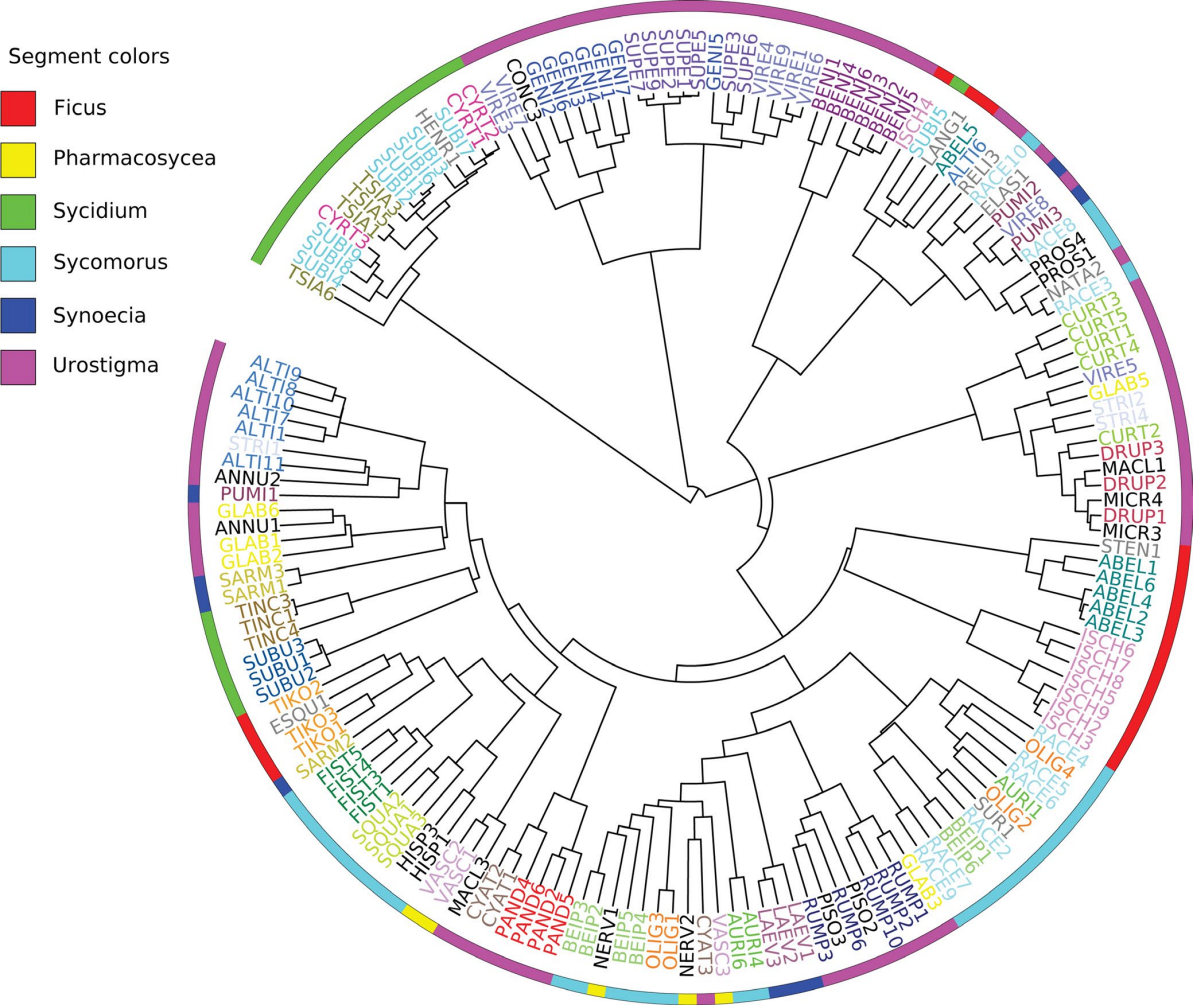
Results of hierarchical clustering showed that most or all individuals of a given species frequently share similar FEF assemblages. Species represented by one individual are shown in gray, species with two individuals are shown in black, and species represented by more than two individuals are each given unique colors. Six subgenera are marked by segments of different colors on the rim of the circle. The numbers after the abbreviations denote the different individuals for a given species

The results of both the ANOSIM and PERMANOVA analyses showed that FEF assemblages differed significantly among host species in each of the three groups including subsection *Conocycea*, subsection *Urostigma*, and the *F. auriculata* species complex (Table 1). Pairwise comparisons of multivariate dispersion showed that the variation in FEF assemblages was heterogeneous in subsections *Conocycea* (Table S4) and *Urostigma* (Table S5), and homogeneous in the *F. auriculata* species complex (Table S6). DCA ordination revealed the dissimilarity among FEF assemblages of closely related host species from different groups, which also included subsection *Conocycea* (Figure 1a), subsection *Urostigma* (Figure 1b), and the *F. auriculata* species complex (Figure 1c). Hierarchical clustering (Figure 2) showed that on average FEF assemblages from 80% of *Ficus* individuals clustered together for host species with two or more individuals (height value in hierarchical clustering was arbitrarily set to one to identify separate groups), especially the FEF assemblages of all individuals for 13 host species clustered in separate groups.

**Table 1:** Host identity contributed significantly to explaining the structure of FEF assemblages across three groups of closely related species. Both ANOSIM (analysis of similarities) and PERMANOVA (permutational multivariate analysis of variance) were used to detect differences among groups. Permutation tests of multivariate homogeneity of dispersions were conducted for each group before PERMANOVA. Results of the global comparison are given in the column “Homogeneity”; results of pairwise comparisons are given in Tables S3–S5

	ANOSIM		PERMANOVA			Homogeneity	
	*R*	*p*	*R* ^2^	*F*	*p*	*F*	*p*
*F.auriculata* species complex	0.19	**.032**	0.29	2.00	**.005**	0.41	.754
Subsection *Conosycea*	0.83	**.001**	0.67	9.44	**.001**	2.76	**.037**
Subsection *Urostigma*	0.53	**.001**	0.55	9.41	**.001**	3.57	**.027**

Note

*P* values < 0.05 are shown in bold.

### 3.2 Host plant phylogenetic relatedness and similarity of FEF assemblages

**Figure 3:**
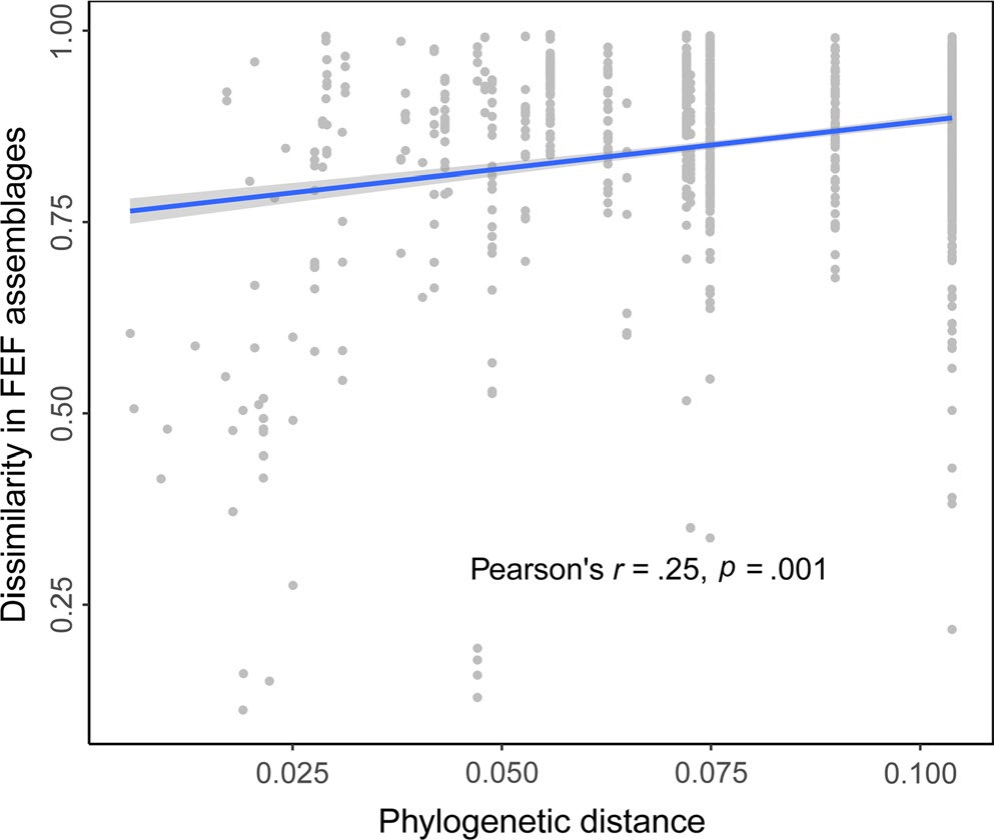
Positive correlation between phylogenetic distance and dissimilarity between the FEF assemblages of 46 *Ficus* species in common‐garden conditions (Confidence level = 0.95)

The Mantel test showed that the dissimilarity in FEF assemblages was significantly correlated with the phylogenetic distance separating host plants (*r* = .25, *p* = .001, *n* = 46; Figure 3). The six subgenera were characterized by significantly different FEF assemblages (ANOSIM, *R* = 0.32, *p* = .001, Figure S3).

### 3.3 Leaf traits and the composition of FEF assemblages

**Figure 4:**
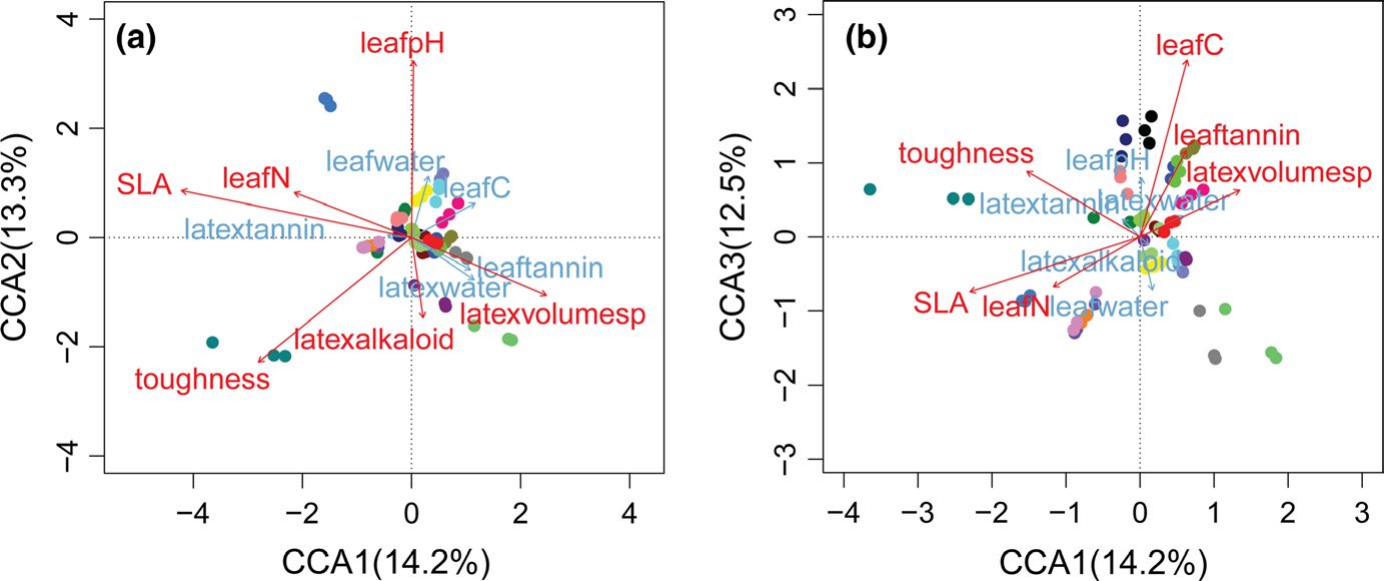
Two‐dimensional CCA ordination, showing that leaf traits contribute to explaining the dissimilarity among FEF assemblages of the 23 *Ficus* species represented by three individuals (a: ordination space of CCA 1 and CCA2; b: CCA 1 and CCA3, leaf carbon content contributed more to CCA3 than to other axes). Variables with significant effects (*p* < .05 by “envfit” function in vegan) are colored red. Each species are given unique colors; points refer to each of the 69 individuals. Latexvolumesp refers to latex volume per leaf area

The CCA was used to test and illustrate whether leaf traits mediate the variation in FEF assemblages (Figure 4). In total, 32.9% of the variation was explained by the 11 traits. CCA1 and CCA2 together accounted for 27.5% of the cumulative proportion of the variation, and six traits (SLA, leaf N content, leaf pH, toughness, latex alkaloid content, and latex volume per leaf area) were significant when fitted to CCA1 and CCA2 (Figure 4a). Leaf C content contributed more to CCA3 than to the first two axes (Figure 4b). Among the 11 traits measured, only leaf C showed a significant phylogenetic signal (Blomberg's *K* = 1.2, *p* < .001). The other 10 traits did not present significant phylogenetic signals (Table S7).

We determined which single leaf trait could predict the similarity between FEF assemblages. The Euclidean distance of SLA, leaf pH, and toughness was significantly correlated with the similarity among FEF assemblages (Table 2), both before and after controlling for the impact of host phylogeny. In the multiple regression on distance matrix analysis, 21.0% of the total variance in FEF assemblage was explained by both host phylogeny and leaf traits while phylogeny alone explained 7.9%, which is a considerable proportion of the total variance. Among the different leaf traits, SLA and leaf pH had a significant impact on FEF assemblages (Table 3).

**Table 2:** Correlation between similarity of FEF assemblages and Euclidian distance of each leaf trait, using partial Mantel test to control the impact of host phylogeny. Species number = 23; OTUs were pooled by sum and then transformed to proportion of the total reads count in each sample

Predictor	Simple Mantel test		Partial Mantel test	
	*r*	*p*	*r*	*p*
SLA (cm^2^/g dry mass)	.238	**.006**	.265	**.001**
Leaf water content (%)	−.070	.432	−.080	.372
Leaf C content (g/kg)	.118	.127	−.042	.565
Leaf N content (g/kg)	−.025	.774	−.016	.852
Leaf pH	.230	**.005**	.247	**.002**
Toughness (g)	.189	**.035**	.214	**.018**
Leaf tannin content per mass (%)	−.008	.940	−.038	.665
Latex volume per area (μl/cm^2^)	.039	.640	.024	.789
Latex water content (%)	−.021	.794	−.010	.895
Latex alkaloid content (g/L)	−.124	.103	−.109	.156
Latex tannin content (g/L)	−.105	.209	−.127	.119

Note

*P* values < 0.05 are shown in bold.

**Table 3:** Multiple regression on distance matrix revealed that host phylogeny, SLA, and leaf pH significantly impact the FEF assemblages (*R* ^2^ = 0.21, *p* = .0001; *F* = 5.33, *p* = .0001). The fraction of variation purely explained by each predictor was presented as percentage in the last column

Predictors	Regression coefficient	*p*	Percentage of variation purely explained (%)
Intercept	0.709	.998	
Host phylogeny	1.435	**.001**	7.9
SLA	0.031	**.004**	4.9
Leaf water content	−0.009	.427	0.3
Leaf C content	−0.004	.782	0.1
Leaf N content	0.004	.738	0.1
Leaf pH	0.026	**.011**	3.9
Toughness	0.010	.340	0.7
Leaf tannin content per mass	0.002	.848	0.0
Latex volume per area	−0.005	.675	0.1
Latex water content	0.001	.946	0.0
Latex alkaloid content	−0.003	.808	0.0
Latex tannin content	−0.006	.627	0.1

Note

*P* values < 0.05 are shown in bold.

## 4 DISCUSSION

This study provides evidence that both host species identity and phylogenetic signal play a significant role in the assemblages of FEF in different *Ficus* species and that the leaf traits studied explained a considerable proportion of the variation in FEF assemblages among host species. Thus, this study suggests that both evolutionary and ecological processes shape FEF assemblages in *Ficus*.

Different host species harbored different FEF assemblages (Figure 1, Table 1; Figure 2). This was true for even very closely related species of the *F. auriculata* complex. While genetically distinct (Wei, Kobmoo, Cruaud, & Kjellberg, 2014), they are similar morphologically (Berg, 2007) and sometimes even share pollinators (Wang, Cannon Charles, & Chen, 2016; Yang, Li, Peng, & Yang, 2012). ANOSIM and PERMANOVA analyses showed that FEF assemblages of the four species in this complex differed from each other. More distinct FEF assemblages were detected in species in the well‐supported subsections *Conocycea* and *Urostigma*. For each *Ficus* species sampled in this study, different individuals were usually characterized by very similar FEF assemblages (Figure 2). This result is consistent with that of previous culture‐based studies (Dastogeer et al., 2018; Solis et al., 2016; Vincent et al., 2016), in which more phylogenetically distant hosts were included. Considering all the evidence, we conclude that host identity is a key factor shaping the structure of FEF assemblages, especially for host species sharing a similar habitat.

The Mantel test revealed a significant correlation between FEF similarity and the phylogenetic proximity of host species (Figure 3). A phylogenetic signal refers to the tendency of evolutionarily related organisms to resemble each other (Blomberg et al., 2003). Descendants inherit the resemblance from a common ancestor and show phenotypic stability even within a set of characteristics performing certain functions (Schwenk & Wagner, 2001). The phylogenetic signal found in this study contributes to shaping FEF assemblages could be generally interpreted as the result of phylogenetic conservatism in maintaining suites of fungi hosted among plants (Gilbert & Webb, 2007; Shefferson et al., 2010). Our results contrast with those of a recent culture‐based study (Vincent et al., 2016), in which a Mantel test did not show a significant correlation between FEF assemblages and host phylogeny. One possible explanation for this difference might be that not all fungal species can be cultured (Hyde & Soytong, 2008), although other plausible explanations do exist.

The distinct chemical composition and physical traits of leaves might underlie the influence of hosts on FEF assemblages. In this study, 32.9% of the variation in FEF assemblages was explained by the 11 leaf traits studied. This result is consistent with that of the culture‐based study by Vincent et al. (2016), in which the variation explained by three leaf traits ranged from 11% to 22% at different sampling sites. Arnold et al. (2003) found that host leaf extracts compared with leaf extracts of nonhosts significantly enhanced the growth of FEF that had abundantly colonized the tested host and that the frequently isolated endophytes consistently succeeded in interacting with relatively rare endophytes in a medium containing leaf extracts. This suggests that host‐specific leaf chemistry may mediate the interactions between endophytes, thereby influencing endophyte species composition. Among the 11 leaf traits tested in this study, only leaf carbon content showed a significant phylogenetic signal (Table S7), suggesting that there must be other phylogenetic constrained traits contributing to FEF assemblages. SLA, leaf pH, and toughness did not present phylogenetic signals; however, the simple Mantel test and partial Mantel test (Table 2) revealed that they were significantly able to predict similarities among FEF assemblages. In addition, only SLA and leaf pH were significant in the multiple regression on distance matrix analysis (Table 3). Nevertheless, these results provide evidence that species‐specific leaf traits affect FEF assemblages.

Although most fig species included in this study are native to the Yunnan province in China, many of them were introduced into the botanical garden as seedlings collected from different sites in the wild. Thus, there is a possibility that FEF recruited from native habitats have influenced the FEF assemblages in our study. However, the influence might be extremely limited. *Ficus* individuals have been introduced into the garden for at least four years (Table S1), providing enough time for the host to recruit horizontally transmitted (Rodriguez et al., 2009) FEF from the new environment. Moreover, the figs introduced into the garden were cultivated in conditions that partially modeled their natural habitat preferences. However, as our study was limited by an extremely unbalanced sample size, the influence of microhabitat on FEF assemblages was not assessed.

More than 5,000 nonsingleton OTUs were observed from 46 *Ficus* species, suggesting that FEF are very diverse in the garden, which is consistent with findings in the tropics (Arnold, 2007; Zimmerman & Vitousek, 2012). However, the OTU‐based approach cannot address the question of whether particular FEF species play important roles in their interactions with host plants (Lieckfeldt & Seifert, 2000). In this case, a culture‐based approach combined with observations under controlled conditions may shed new light on understanding the ecological functions of FEF in host plants.

Host‐mediated filtering is the cornerstone of research on plant–FEF interactions. By characterizing FEF assemblages of 46 congeneric species in a small area, and thereby minimizing the variation in abiotic environmental conditions and fungal dispersal capacity, this study provides strong evidence that host identity has an important influence on shaping FEF assemblages and that leaf traits contribute to explaining the effect of host identity. Host phylogeny was also found to exert a significant impact on shaping FEF assemblages. Our findings suggest that both evolutionary and ecological processes play a role in shaping FEF assemblages, which is fundamental to understanding the mechanisms for FEF assemblage establishment.

## CONFLICT OF INTEREST

Authors declare no conflict of interest.

## AUTHOR CONTRIBUTIONS

J. L. and J. C. designed the study, J. L. and J. Z. collected the data, J. L. performed the statistical analyses, J. L. and J. C. drafted the manuscript, and all the authors approved the final version.

## Data Availability

DNA sequences: SRR8675934 (SRA, NCBI).
