# Supplementary material for: Host identity and phylogeny shape the foliar endophytic fungal assemblages of Ficus
Source: Ecol Evol. 2019 Aug 13;9(18):10472–82. doi: 10.1002/ece3.5568 (PMC6787831; doi:10.1002/ece3.5568)
Supplement: Supplementary file 1 [file ECE3-9-10472-s001.docx]

**Title:** Host identity and phylogeny shape the foliar endophytic fungal assemblages of *Ficus*

Junwei Liu^1, 2^, Jin Zhao^1^, Gang Wang^1^ and Jin Chen^1^

^1^CAS Key Laboratory of Tropical Forest Ecology, Xishuangbanna Tropical Botanical Garden, Chinese Academy of Sciences, Mengla, Yunnan 666303, China

^2^University of Chinese Academy of Sciences, Beijing 100049, China

**Supplementary information**

Table S1: Provenance, nature of initially introduced propagules and sample numbers for study species in the living *Ficus* collection at XTBG (46 species, 166 individuals).

| Latin name | Provenance | Date | Ways for propagation | Subgenus | Abbreviations | Sample number | Total samples |
| --- | --- | --- | --- | --- | --- | --- | --- |
| *F. abelii* | Tianlin, Guangxi, China | 12-05-2007 | Seedlings from wild | Ficus | ABEL | 6 | 6 |
| *F. altissima* | No information | No information | No information | Urostigma | ALTI | 4 | 7 |
|  | Yingjiang, Yunnan, China | 04-01-1994 | No information | Urostigma |  | 3 |  |
| *F. annulata* | Mengla, Yunnan, China | 12-29-2006 | Seedlings from wild | Urostigma | ANNU | 1 | 2 |
|  | No information | No information | No information | Urostigma |  | 1 |  |
| *F. auriculata* | Jinghong, Yunnan, China | 05-27-2008 | No information | Sycomorus | AURI | 1 | 3 |
|  | Yingjiang, Yunnan, China | 04-02-1994 | Seedlings from wild | Sycomorus |  | 2 |  |
| *F. beipeiensis* | Chishui, Guizhou, China | 05-15-2007 | Seeds | Sycomorus | BEIP | 6 | 6 |
| *F. benjamina* | Cambodia | 04-11-2008 | Seeds | Urostigma | BENJ | 6 | 6 |
| *F. concinna* | Yingjiang, Yunnan, China | 04-02-1994 | Seedlings from wild | Urostigma | CONC | 1 | 1 |
| *F. curtipes* | Mengla, Yunnan, China | 02-16-1994 | Seedlings from wild | Urostigma | CURT | 5 | 5 |
| *F. cyathistipula* | Lingshui, Hainan, China | 10-25-2001 | Seedlings from wild | Urostigma | CYAT | 3 | 3 |
| *F. cyrtophylla* | Yingjiang, Yunnan, China | 04-01-1994 | Seedlings from wild | Sycidium | CYRT | 3 | 3 |
| *F. drupacea* | Yingjiang, Yunnan, China | 04-28-2011 | Cutting | Urostigma | DRUP | 3 | 3 |
| *F. elastica* | No information | No information | No information | Urostigma | ELAS | 1 | 1 |
| *F. esquiroliana* | Mengla, Yunnan, China | 03-26-1994 | Seedlings from wild | Ficus | ESQU | 1 | 1 |
| *F. fistulosa* | Mengla, Yunnan, China | 11-11-2008 | Seedlings from wild | Sycomorus | FIST | 4 | 4 |
| *F. geniculata* | Mengla, Yunnan, China | 01-25-2007 | Seeds | Urostigma | GENI | 7 | 7 |
| *F. glaberrima* | Funing, Yunnan, China | 12-05-2007 | No information | Urostigma | GLAB | 1 | 5 |
|  | Menglian, Yunnan, China | 04-28-2011 | Cutting | Urostigma |  | 1 |  |
|  | Motuo, Tibet, China | 10-01-2003 | No information | Urostigma |  | 1 |  |
|  | Yingjiang, Yunnan, China | 06-03-1997 | Seedlings from wild | Urostigma |  | 2 |  |

Table S1: Continued.

| Latin name | Provenance | Date | Ways for propagation | Subgenus | Abbreviations | Sample number | Total samples |
| --- | --- | --- | --- | --- | --- | --- | --- |
| *F. henryi* | Ruili, Yunnan, China | 07-10-2005 | Seeds | Sycidium | HENR | 1 | 1 |
| *F. hispida* | Yingjiang, Yunnan, China | 04-01-1994 | Seedlings from wild | Sycomorus | HISP | 2 | 2 |
| *F. ischnopoda* | Mengla, Yunnan, China | 05-17-1996 | Seedlings from wild | Ficus | ISCH | 8 | 8 |
| *F. laevis* | Jiangcheng, Yunnan, China | 04-30-2009 | No information | Synoecia | LAEV | 2 | 3 |
|  | Xiangming, Yunnan, China | 11-06-2007 | Cutting | Synoecia |  | 1 |  |
| *F. langkokensis* | Mengla, Yunnan, China | 05-17-1996 | Seedlings from wild | Ficus | LANG | 1 | 1 |
| *F. maclellandi* | Ankang, Shanxi, China | 12-15-1995 | Seedlings from wild | Urostigma | MACL | 2 | 2 |
| *F. microcarpa* | Lvchun, Yunnan, China | 12-07-1997 | Seedlings from wild | Urostigma | MICR | 2 | 2 |
| *F. natalensis* | Lingshui, Hainan, China | 10-05-2001 | Seedlings from wild | Urostigma | NATA | 1 | 1 |
| *F. nervosa* | Daxin, Guangxi, China | 11-03-2002 | Seedlings from wild | Pharmacosycea | NERV | 1 | 2 |
|  | Honghe, Yunnan, China | 11-28-2001 | Seedlings from wild | Pharmacosycea | NERV | 1 |  |
| *F. oligodon* | Yingjiang, Yunnan, China | 04-02-1994 | Seedlings from wild | Sycomorus | OLIG | 4 | 4 |
| *F. pandurata* | Ruili, Yunnan, China | 05-31-1997 | Seedlings from wild | Urostigma | PAND | 4 | 4 |
| *F. pisocarpa* | Jinghong, Yunnan, China | 07-11-2007 | Seedlings from wild | Urostigma | PISO | 2 | 2 |
| *F. prostrata* | Motuo, Tibet, China | 10-01-2003 | Seeds | Sycomorus | PROS | 2 | 2 |
| *F. pumila* | Pingxiang, Guangxi, China | 07-23-2002 | Cutting | Synoecia | PUMI | 3 | 3 |
| *F. racemosa* | Laos | 24-02-2002 | No information | Sycomorus | RACE | 3 | 9 |
|  | Vietnam | 01-18-2001 | No information | Sycomorus |  | 3 |  |
|  | Vietnam | 01-19-2001 | No information | Sycomorus |  | 3 |  |
| *F. religiosa* | Mengla, Yunnan, China | 03-23-1994 | Seedlings from wild | Urostigma | RELI | 1 | 1 |
| *F. rumphii* | Vietnam | 01-19-2001 | Seeds | Urostigma | RUMP | 5 | 5 |
| *F. sarmentosa* | Mengla, Yunnan, China | 12-17-2000 | Seedlings from wild | Synoecia | SARM | 3 | 3 |
| *F. squamosa* | Mengla, Yunnan, China | 05-17-1996 | Seedlings from wild | Sycomorus | SQUA | 3 | 3 |
| *F. stenophylla* | Menglian, Yunnan, China | 08-15-2007 | Cutting | Ficus | STEN | 1 | 1 |

Table S1: Continued.

| Latin name | Provenance | Date | Ways for propagation | Subgenus | Abbreviations | Sample number | Total samples |
| --- | --- | --- | --- | --- | --- | --- | --- |
| *F. stricta* | Yingjiang, Yunnan, China | 04-01-1994 | Seedlings from wild | Urostigma | STRI | 3 | 3 |
| *F. subincisa* | Motuo, Tibet, China | 10-01-2003 | Seeds | Sycidium | SUBI | 4 | 9 |
|  | Motuo, Tibet, China | 10-01-2004 | Seeds | Sycidium |  | 2 |  |
|  | Yingjiang, Yunnan, China | 06-01-2002 | Seedlings from wild | Sycidium |  | 3 |  |
| *F. subulata* | Mengla, Yunnan, China | 08-18-2008 | Seeds | Sycidium | SUBU | 3 | 3 |
| *F. superba* | Haikou, Hainan, China | 11-10-2002 | Seeds | Urostigma | SUPE | 3 | 7 |
|  | Menghai, Yunnan, China | 06-20-2009 | Seedlings from wild | Urostigma |  | 4 |  |
| *F. sur* | South Africa | 10-29-2001 | Seeds | Sycomorus | SUR | 1 | 1 |
| *F. tikoua* | Qiaojia, Yunnan, China | 07-27-2004 | Cutting | Ficus | TIKO | 3 | 3 |
| *F. tinctoria* | Thailand | 11-11-2002 | No information | Sycidium | TINC | 3 | 3 |
| *F. tsiangii* | Wenshan, Yunnan, China | 09-11-2001 | Seeds | Sycidium | TSIA | 4 | 4 |
| *F. vasculosa* | Jianfengling, Hainan, China | 09-18-2008 | Seeds | Pharmacosycea | VASC | 3 | 3 |
| *F. virens* | Funing, Yunnan, China | 12-05-2007 | Seedlings from wild | Urostigma | VIRE | 8 | 8 |

Table S2: Values of the 11 measured leaf traits for three selected individuals of the 23 *Ficus* species represented in the collection by at least this number of individuals.

| Individual code | Latin name | Leaf pH | SLA (cm^2^ ∙ g^-1^) | Leaf C (g∙kg^-1^) | Leaf N (g∙kg^-1^) | Toughness (g) | Leaf water (%) | Leaf tannin (g∙L^-1^) | Latex volume per area (μl∙cm^-2^) | Latex water (%) | Latex tannin (g∙L^-1^) | Latex alkaloid (g∙L^-1^) |
| --- | --- | --- | --- | --- | --- | --- | --- | --- | --- | --- | --- | --- |
| SUBU-01 | *F. subulata* | 8.42 | 458.01 | 430.00 | 23.68 | 65.34 | 85.75 | 1.74 | 0.05 | 81.82 | 8.94 | 0.36 |
| SUBU-02 | *F. subulata* | 9.28 | 460.59 | 430.00 | 24.36 | 92.57 | 87.18 | 1.86 | 0.07 | 77.59 | 7.56 | 0.37 |
| SUBU-03 | *F. subulata* | 8.46 | 538.91 | 432.00 | 26.18 | 66.35 | 88.81 | 1.49 | 0.05 | 71.19 | 6.41 | 0.35 |
| ABEL-01 | *F. abelii* | 6.55 | 168.56 | 426.00 | 17.50 | 39.83 | 78.24 | 2.09 | 0.54 | 80.95 | 14.27 | 3.53 |
| ABEL-02 | *F .abelii* | 6.77 | 165.64 | 426.00 | 18.33 | 58.36 | 72.60 | 1.98 | 0.41 | 81.25 | 13.29 | 3.48 |
| ABEL-03 | *F. abelii* | 6.52 | 160.83 | 428.00 | 18.56 | 30.57 | 85.74 | 2.14 | 0.95 | 89.29 | 15.21 | 3.48 |
| ALTI-07 | *F. altissima* | 8.11 | 102.96 | 510.54 | 25.37 | 89.68 | 77.11 | 2.20 | 0.63 | 84.93 | 16.12 | 3.50 |
| ALTI-08 | *F .altissima* | 7.86 | 118.41 | 501.00 | 26.31 | 90.00 | 79.32 | 1.98 | 0.73 | 86.07 | 17.63 | 3.50 |
| ALTI-09 | *F .altissima* | 7.71 | 131.51 | 506.39 | 24.34 | 101.25 | 81.99 | 2.00 | 0.52 | 84.76 | 17.24 | 3.52 |
| CURT-01 | *F .curtipes* | 8.69 | 121.28 | 481.26 | 10.37 | 102.65 | 71.84 | 5.70 | 0.38 | 74.22 | 3.29 | 1.91 |
| CURT-04 | *F. curtipes* | 8.82 | 139.20 | 482.36 | 12.05 | 125.84 | 79.61 | 5.99 | 0.57 | 67.61 | 4.23 | 1.89 |
| CURT-05 | *F. curtipes* | 8.32 | 112.43 | 480.25 | 11.25 | 131.75 | 75.09 | 6.10 | 0.59 | 77.88 | 3.83 | 1.90 |
| GENI-01 | *F. geniculata* | 7.31 | 352.71 | 445.00 | 23.30 | 59.55 | 91.05 | 1.90 | 0.48 | 75.00 | 21.57 | 1.53 |
| GENI-02 | *F. geniculata* | 6.25 | 329.89 | 436.00 | 22.55 | 71.53 | 83.71 | 1.99 | 0.21 | 77.14 | 22.57 | 1.51 |
| GENI-03 | *F .geniculata* | 6.97 | 332.48 | 439.00 | 23.35 | 54.93 | 80.36 | 1.98 | 0.20 | 79.41 | 20.62 | 1.54 |
| PAND-04 | *F. pandurata* | 8.65 | 105.02 | 460.00 | 14.56 | 90.98 | 73.99 | 3.05 | 0.22 | 82.72 | 7.59 | 0.63 |
| PAND-05 | *F. pandurata* | 8.50 | 101.84 | 462.00 | 15.35 | 101.05 | 79.16 | 4.36 | 0.25 | 87.45 | 8.17 | 0.62 |
| PAND-06 | *F. pandurata* | 7.67 | 101.04 | 463.00 | 14.84 | 106.42 | 72.84 | 3.95 | 0.19 | 85.24 | 5.65 | 0.64 |
| BENJ-01 | *F. benjamina* | 6.31 | 306.19 | 455.00 | 17.89 | 114.94 | 84.69 | 0.79 | 0.32 | 75.00 | 4.70 | 1.39 |
| BENJ-02 | *F. benjamina* | 8.72 | 261.36 | 450.00 | 17.68 | 156.77 | 83.07 | 0.85 | 0.26 | 72.73 | 5.28 | 1.39 |
| BENJ-03 | *F. benjamina* | 8.98 | 267.41 | 452.00 | 17.94 | 129.74 | 84.85 | 0.79 | 0.36 | 77.14 | 3.88 | 1.39 |
| AURI-01 | *F. auriculata* | 7.65 | 138.92 | 459.00 | 21.25 | 71.00 | 85.19 | 3.49 | 0.72 | 81.35 | 2.50 | 3.17 |

Table S2: Continued.

| Individual code | Latin name | Leaf pH | SLA (cm^2^ ∙ g^-1^) | Leaf C (g∙kg^-1^) | Leaf N (g∙kg^-1^) | Toughness (g) | Leaf water (%) | Leaf tannin (g∙L^-1^) | Latex volume per area (μl∙cm^-2^) | Latex water (%) | Latex tannin (g∙L^-1^) | Latex alkaloid (g∙L^-1^) |
| --- | --- | --- | --- | --- | --- | --- | --- | --- | --- | --- | --- | --- |
| AURI-04 | *F. auriculata* | 7.71 | 148.35 | 458.00 | 20.50 | 77.00 | 82.08 | 4.06 | 0.62 | 76.98 | 2.09 | 3.16 |
| AURI-06 | *F. auriculata* | 8.35 | 119.59 | 457.00 | 22.36 | 75.00 | 82.35 | 3.79 | 0.48 | 78.17 | 2.20 | 3.16 |
| SUPE-01 | *F. superba* | 9.47 | 213.29 | 446.00 | 14.23 | 55.00 | 76.67 | 2.49 | 0.07 | 50.00 | 11.67 | 1.31 |
| SUPE-02 | *F. superba* | 8.66 | 198.89 | 445.00 | 13.69 | 78.00 | 71.88 | 2.68 | 0.07 | 66.67 | 9.76 | 1.32 |
| SUPE-03 | *F. superba* | 9.65 | 199.00 | 443.00 | 13.67 | 84.00 | 75.00 | 2.74 | 0.06 | 64.52 | 10.46 | 1.30 |
| SUBI-01 | *F. subincisa* | 7.99 | 305.33 | 418.00 | 19.23 | 107.00 | 68.42 | 1.26 | 0.04 | 59.00 | 10.31 | 4.46 |
| SUBI-02 | *F. subincisa* | 8.80 | 347.25 | 418.00 | 18.45 | 102.00 | 81.82 | 1.98 | 0.06 | 65.48 | 10.53 | 4.46 |
| SUBI-04 | *F. subincisa* | 7.26 | 284.30 | 416.00 | 18.27 | 110.00 | 70.59 | 1.70 | 0.03 | 69.44 | 10.90 | 4.52 |
| VIRE-01 | *F. virens* | 8.88 | 180.00 | 440.00 | 18.54 | 59.00 | 80.00 | 3.79 | 0.24 | 83.10 | 11.02 | 3.39 |
| VIRE-04 | *F. virens* | 8.65 | 186.25 | 440.00 | 17.36 | 57.00 | 83.33 | 3.49 | 0.35 | 74.00 | 11.34 | 3.35 |
| VIRE-06 | *F. virens* | 8.88 | 185.75 | 442.00 | 16.57 | 58.00 | 84.62 | 3.06 | 0.35 | 80.77 | 11.32 | 3.45 |
| SARM-01 | *F. sarmentosa* | 5.75 | 84.06 | 420.00 | 11.23 | 65.34 | 81.57 | 4.06 | 0.54 | 82.84 | 1.02 | 2.32 |
| SARM-02 | *F. sarmentosa* | 5.22 | 87.10 | 422.00 | 13.23 | 92.57 | 81.33 | 4.29 | 0.57 | 83.96 | 0.98 | 2.37 |
| SARM-03 | *F. sarmentosa* | 5.49 | 89.10 | 425.00 | 12.56 | 66.35 | 82.49 | 4.90 | 0.47 | 82.84 | 1.70 | 2.28 |
| STRI-01 | *F. stricta* | 8.20 | 74.13 | 478.00 | 13.56 | 81.00 | 57.89 | 7.92 | 0.58 | 73.83 | 45.25 | 5.52 |
| STRI-02 | *F. stricta* | 9.30 | 70.29 | 479.00 | 12.33 | 79.00 | 44.00 | 7.59 | 0.57 | 74.94 | 50.03 | 5.39 |
| STRI-04 | *F. stricta* | 7.34 | 74.30 | 480.00 | 13.23 | 81.00 | 52.38 | 7.49 | 0.54 | 75.22 | 37.21 | 5.49 |
| DRUP-01 | *F. drupacea* | 9.16 | 165.69 | 470.00 | 17.26 | 213.00 | 87.13 | 1.59 | 1.07 | 77.57 | 14.57 | 1.99 |
| DRUP-02 | *F. drupacea* | 9.36 | 167.73 | 476.00 | 16.57 | 203.00 | 85.15 | 1.09 | 1.19 | 74.79 | 14.18 | 1.98 |
| DRUP-03 | *F. drupacea* | 8.82 | 166.53 | 478.00 | 17.45 | 223.00 | 86.43 | 0.78 | 1.32 | 76.77 | 14.52 | 1.99 |
| CYRT-01 | *F. cyrtophylla* | 7.15 | 171.17 | 412.00 | 38.00 | 46.00 | 79.31 | 1.49 | 0.44 | 78.20 | 20.66 | 1.29 |
| CYRT-02 | *F. cyrtophylla* | 9.26 | 192.79 | 413.00 | 38.59 | 50.00 | 78.46 | 1.06 | 0.34 | 80.38 | 20.20 | 1.28 |
| CYRT-03 | *F. cyrtophylla* | 8.12 | 179.90 | 410.00 | 37.56 | 48.00 | 77.27 | 1.57 | 0.51 | 87.05 | 20.29 | 1.29 |

Table S2: Continued.

| Individual code | Latin name | Leaf pH | SLA (cm^2^ ∙ g^-1^) | Leaf C (g∙kg^-1^) | Leaf N (g∙kg^-1^) | Toughness (g) | Leaf water (%) | Leaf tannin (g∙L^-1^) | Latex volume per area (μl∙cm^-2^) | Latex water (%) | Latex tannin (g∙L^-1^) | Latex alkaloid (g∙L^-1^) |
| --- | --- | --- | --- | --- | --- | --- | --- | --- | --- | --- | --- | --- |
| FIST-03 | *F. fistulosa* | 8.21 | 223.88 | 449.00 | 19.79 | 95.00 | 83.33 | 5.91 | 0.07 | 73.53 | 16.48 | 2.20 |
| FIST-04 | *F. fistulosa* | 9.01 | 212.08 | 440.00 | 24.36 | 88.00 | 86.05 | 5.57 | 0.10 | 77.58 | 14.23 | 2.20 |
| FIST-05 | *F. fistulosa* | 8.57 | 225.00 | 445.00 | 19.58 | 81.00 | 85.71 | 5.69 | 0.12 | 78.30 | 15.25 | 2.21 |
| TSIA-03 | *F. tsiangii* | 8.30 | 336.94 | 399.00 | 24.56 | 152.00 | 78.57 | 2.91 | 0.05 | 70.41 | 15.02 | 1.79 |
| TSIA-05 | *F. tsiangii* | 8.38 | 365.42 | 408.00 | 25.98 | 165.00 | 77.38 | 2.13 | 0.05 | 66.67 | 17.65 | 1.85 |
| TSIA-06 | *F. tsiangii* | 8.30 | 350.45 | 406.00 | 26.14 | 145.00 | 80.00 | 2.29 | 0.07 | 69.61 | 17.41 | 1.81 |
| PUMI-01 | *F. pumila* | 6.32 | 688.00 | 433.00 | 18.25 | 316.00 | 85.71 | 2.30 | 0.04 | 77.27 | 14.26 | 3.00 |
| PUMI-02 | *F. pumila* | 6.44 | 441.50 | 430.00 | 18.36 | 320.00 | 50.00 | 2.16 | 0.04 | 68.33 | 15.66 | 3.01 |
| PUMI-03 | *F. pumila* | 6.23 | 418.50 | 432.00 | 18.26 | 312.00 | 71.43 | 2.20 | 0.04 | 77.27 | 12.62 | 3.05 |
| ISCH-02 | *F. ischnopoda* | 6.34 | 166.00 | 442.00 | 18.33 | 71.00 | 86.36 | 8.00 | 0.32 | 76.42 | 10.74 | 0.78 |
| ISCH-03 | *F. ischnopoda* | 6.64 | 160.00 | 446.00 | 21.14 | 78.00 | 76.92 | 8.16 | 0.32 | 68.91 | 8.93 | 0.73 |
| ISCH-08 | *F. ischnopoda* | 6.42 | 158.75 | 440.00 | 19.36 | 69.00 | 78.95 | 7.98 | 0.30 | 70.67 | 6.59 | 0.78 |
| GLAB-01 | *F. glaberrima* | 8.40 | 122.16 | 480.00 | 20.36 | 87.64 | 67.45 | 12.36 | 0.25 | 88.13 | 8.37 | 0.12 |
| GLAB-02 | *F. glaberrima* | 8.41 | 109.07 | 482.00 | 21.50 | 81.02 | 73.36 | 12.84 | 0.20 | 82.98 | 9.46 | 0.14 |
| GLAB-03 | *F. glaberrima* | 7.81 | 111.60 | 483.00 | 19.63 | 95.20 | 78.67 | 13.05 | 0.26 | 82.38 | 11.83 | 0.14 |
| RUMP-01 | *F. rumphii* | 8.34 | 292.00 | 479.00 | 23.25 | 92.00 | 76.67 | 5.09 | 0.16 | 63.26 | 8.12 | 1.25 |
| RUMP-02 | *F. rumphii* | 7.79 | 234.50 | 475.00 | 21.04 | 85.00 | 72.22 | 4.01 | 0.12 | 66.83 | 9.79 | 1.22 |
| RUMP-03 | *F. rumphii* | 8.13 | 275.88 | 479.00 | 20.56 | 78.00 | 71.43 | 4.26 | 0.14 | 64.32 | 7.26 | 1.27 |
| SQUA-01 | *F. squamosa* | 8.80 | 209.00 | 401.00 | 27.23 | 101.00 | 82.86 | 0.60 | 0.60 | 80.29 | 28.98 | 3.16 |
| SQUA-02 | *F. squamosa* | 8.51 | 240.00 | 404.00 | 27.68 | 122.00 | 87.80 | 0.70 | 0.73 | 85.56 | 29.46 | 3.15 |
| SQUA-03 | *F. squamosa* | 8.58 | 197.50 | 406.00 | 28.36 | 115.00 | 81.40 | 0.81 | 0.43 | 80.71 | 28.12 | 3.17 |
| BEIP-04 | *F. beipeiensis* | 8.51 | 145.69 | 439.00 | 16.33 | 123.00 | 80.60 | 6.80 | 0.27 | 82.58 | 0.54 | 0.22 |

Table S2: Continued.

| Individual code | Latin name | Leaf pH | SLA (cm^2^ ∙ g^-1^) | Leaf C (g∙kg^-1^) | Leaf N (g∙kg^-1^) | Toughness (g) | Leaf water (%) | Leaf tannin (g∙L^-1^) | Latex volume per area (μl∙cm^-2^) | Latex water (%) | Latex tannin (g∙L^-1^) | Latex alkaloid (g∙L^-1^) |
| --- | --- | --- | --- | --- | --- | --- | --- | --- | --- | --- | --- | --- |
| BEIP-05 | *F. beipeiensis* | 8.53 | 124.05 | 437.00 | 15.59 | 116.00 | 81.55 | 6.90 | 0.49 | 83.65 | 0.49 | 0.25 |
| BEIP-06 | *F. beipeiensis* | 9.14 | 118.85 | 425.00 | 17.00 | 96.00 | 83.54 | 7.08 | 0.31 | 76.35 | 0.60 | 0.24 |

Table S3. Species included in the original phylogenetic tree, associated accession numbers for sequences of internal transcribed spacer (ITS), external transcribed spacer (ETS), and glyceraldehyde-3-phosphate dehydrogenase (G3pdh). Two species as outgroup and another two species not sampled were pruned off when conducting phylogenetic analysis.

| **Species** | **ITS** | **G3pdh** | **ETS** | **Note** |
| --- | --- | --- | --- | --- |
| *Antiaropsis decipiens* | AY730142 | EF092326 | EU084403 | Outgroup |
| *Castilla elastica* | AY730143 | KU855601 | AY730232 | Outgroup |
| *F. abelii* | JQ773829 | Na^1^ | KY388629 |  |
| *F. altissima* | AY730064 | EU087621 | AY730152 |  |
| *F. annulata* | EU091578 | EU087622 | EU084417 |  |
| *F. auriculata* | AF165376 | JN117685 | FJ812281 |  |
| *F. beipeiensis* | KM234114 | JN117686 | AY063520 |  |
| *F. benjamina* | AY063559 | JN117687 | Na |  |
| *F. concinna* | AY730059 | JN117692 | AY730145 |  |
| *F. curtipes* | JN117627 | JN117693 | JN117657 |  |
| *F. cyathistipula* | DQ455657 | Na | DQ455679 |  |
| *F. cyrtophylla* | JQ773858 | JN117694 | EU084488 |  |
| *F. drupacea* | AY730066 | EF092335 | AY730154 |  |
| *F. elastica* | HM368192 | EF092338 | AY063516 |  |
| *F. esquiroliana* | JQ773873 | KF811032 | KX055620 |  |
| *F. fistulosa* | AY730137 | JN117695 | KP406992 |  |
| *F. geniculata* | KJ845942 | KJ846000 | KJ845884 |  |
| *F. glaberrima* | JQ773885 | EU087627 | KJ845936 |  |
| *F. henryi* | EU091639 | JN117699 | EU084466 |  |
| *F. hispida* | EU091623 | JN117700 | EU084454 |  |
| *F. ischnopoda* | AY730122 | EF092380 | KY388684 |  |
| *F. laevis* | JQ773913 | Na | Na |  |
| *F. langkokensis* | JN117638 | JN117703 | KY388689 |  |
| *F. maclellandii* | JN117639 | JN117704 | EU084425 |  |
| *F. microcarpa* | JN117640 | JN117705 | JN117665 |  |
| *F. natalensis* | AY730100 | EF092352 | AY730189 |  |
| *F. nervosa* | JQ773926 | EU087615 | EU084410 |  |
| *F. oligodon* | JN117631 | JN117706 | KP406984 |  |
| *F. pandurata* | JQ773945 | Na | KY388713 |  |
| *F. pisocarpa* | JN117643 | JN117707 | JN117667 |  |
| *F. prostrata* | EU091612 | JN117708 | KP406997 |  |
| *F. pumila* | AY063580 | EF092390 | AY063541 |  |
| *F. racemoae* | HM368194 | JN126051 | KP406985 |  |
| *F. religiosa* | JN117645 | EF092331 | AY063543 |  |
| *F. rumphii*  *F. sarmentosa*  *F. squamosa* | AY730063  JQ773982  EU091634 | KJ846039  EU087679  Na | KJ845933  EU084478  Na |  |
|  |  |  |  |  |

Table S3: Continued.

| **Species** | **ITS** | **G3pdh** | **ETS** | **note** |
| --- | --- | --- | --- | --- |
| *F. stenophylla*  *F. stricta* | EU091640  JN117647 | HQ890582  EU087632 | KY388740  EU084429 |  |
| *F. subincisa* | JQ774001 | EU087690 | KY388741 |  |
| *F. subulata* | EU091677 | Na | EU084495 |  |
| *F. superba* | AF165410 | EF092332 | AY730149 |  |
| *F. sur* | AF165411 | EU087649 | KP406986 |  |
| *F. tikoua* | EU091641 | JN117712 | EU084468 |  |
| *F. tinctoria* | AF165413 | JN117713 | AY730223 |  |
| *F. tsiangii* | EU091675 | JN117714 | EU084494 |  |
| *F. vasculosa* | EU091572 | JN117715 | KY388769 |  |
| *F. virens* | JN117616 | JN117684 | AY730150 |  |

^1^Na: Sequences were not obtained.

Table S4. Results of pairwise comparisons in permutation tests of multivariate homogeneity of dispersion showing heterogeneity of dispersion in subsection *Conosycea*. Observed *P*-values are shown below the diagonal, permuted *P*-values above the diagonal. ALTI: *F. altissima*; BENJ: *F. benjamina*; CURT: *F. curtipes*; DRUP: *F. drupacea*; GLAB: *F. glaberrima*; STRI: *F. stricta*. Permuted *P*-values < 0.05 are shown in bold.

|  | ALTI | BENJ | CURT | DRUP | GLAB | STRI |
| --- | --- | --- | --- | --- | --- | --- |
| ALTI |  | 0.100 | 0.177 | 0.065 | 0.480 | 0.917 |
| BENJ | 0.099 |  | 0.680 | 0.457 | **0.024** | 0.256 |
| CURT | 0.161 | 0.684 |  | 0.187 | **0.021** | 0.311 |
| DRUP | 0.059 | 0.454 | 0.175 |  | **0.005** | 0.183 |
| GLAB | 0.460 | 0.019 | 0.017 | 0.008 |  | 0.513 |
| STRI | 0.919 | 0.259 | 0.324 | 0.179 | 0.501 |  |

Table S5. Results of pairwise comparisons in permutation tests of multivariate homogeneity of dispersions showing heterogeneity of dispersion in subsection *Urostigma*. Observed *P*-values are shown below the diagonal, permuted *P*-values above the diagonal. Permuted *P*-values < 0.05 are shown in bold.

|  | *F. geniculata* | *F. rumphii* | *F. superba* | *F. virens* |
| --- | --- | --- | --- | --- |
| *F. geniculata* |  | 0.560 | **0.002** | 0.471 |
| *F. rumphii* | 0.565 |  | **0.004** | 0.780 |
| *F. superba* | 0.003 | 0.006 |  | **0.013** |
| *F. virens* | 0.459 | 0.787 | 0.019 |  |

Table S6. Results of pairwise comparisons in permutation tests of multivariate homogeneity of dispersions showed homogeneity of dispersion in the *F. auriculata* species complex. Observed *P*-values are shown below the diagonal, permuted *P*-values above the diagonal.

|  | *F. auriculata* | *F. beipeiensis* | *F. hainanensis* | *F. oligodon* |
| --- | --- | --- | --- | --- |
| *F. auriculata* |  | 0.614 | 0.634 | 0.906 |
| *F. beipeiensis* | 0.620 |  | 0.816 | 0.239 |
| *F. hainanensis* | 0.615 | 0.832 |  | 0.355 |
| *F. oligodon* | 0.900 | 0.240 | 0.340 |  |

Table S7. Tests for phylogenetic signal of plant traits based on Blomberg’s K statistic for a subset of *Ficus* species (n = 23), using the pruned phylogenetic tree of Figure S1.

| **Traits** | **K** | ***P*** |
| --- | --- | --- |
| SLA (cm^2^∙g^-1^ dry mass) | 0.460 | 0.326 |
| Leaf water content (%) | 0.336 | 0.813 |
| Leaf C content (g∙kg^-1^) | **1.196** | **0.001** |
| Leaf N content (g∙kg^-1^) | 0.478 | 0.353 |
| Leaf pH | 0.472 | 0.350 |
| Toughness (g) | 0.410 | 0.541 |
| Leaf tannin content per mass (%) | 0.537 | 0.157 |
| Latex volume per area (μl∙cm^-2^) | 0.478 | 0.276 |
| Latex water content (%) | 0.454 | 0.310 |
| Latex alkaloid content (g∙L^-1^) | 0.305 | 0.947 |
| Latex tannin content (g∙L^-1^) | 0.369 | 0.729 |

Figure S1. Bayesian phylogenetic tree of *Ficus* inferred from BEAST using sequences of three genes: internal transcribed spacer (ITS), external transcribed spacer (ETS), and glyceraldehyde-3-phosphate-dehydrogenase (G3pdh). *Antiaropsis decipiens* and *Castilla elastica* were used as outgroups. Branch labels refer to posterior probability values. The dendrogram includes all *Ficus* species which were used in the Mantel test (see Fig. 3).


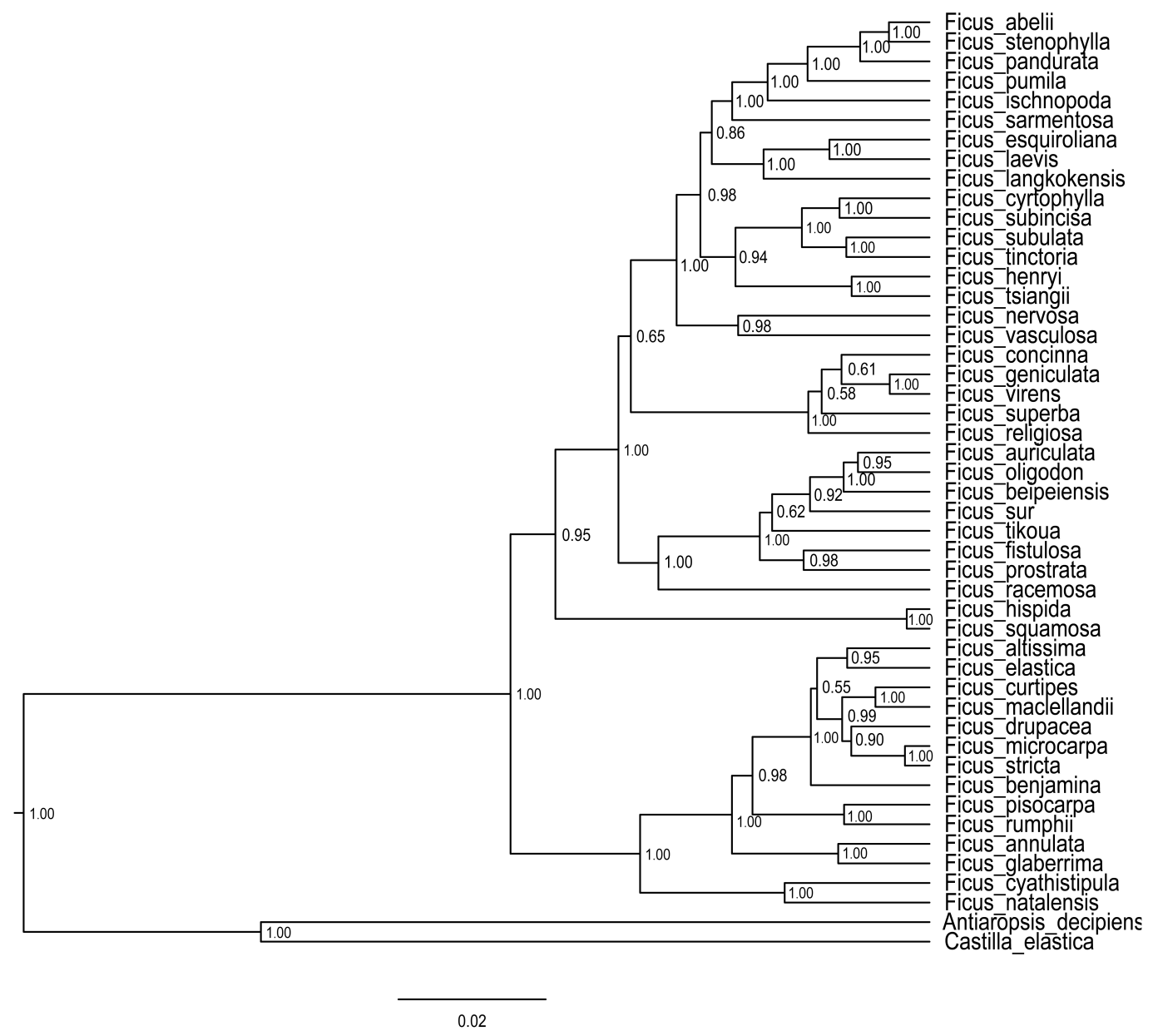


Figure S2. Asymptotic per-tree rarefaction curves of OTU richness showed that sequencing depth was enough to capture the composition of FEF assemblages. OTUs were designated based on 97% similarity of the ITS1 rDNA region and all singletons were removed.


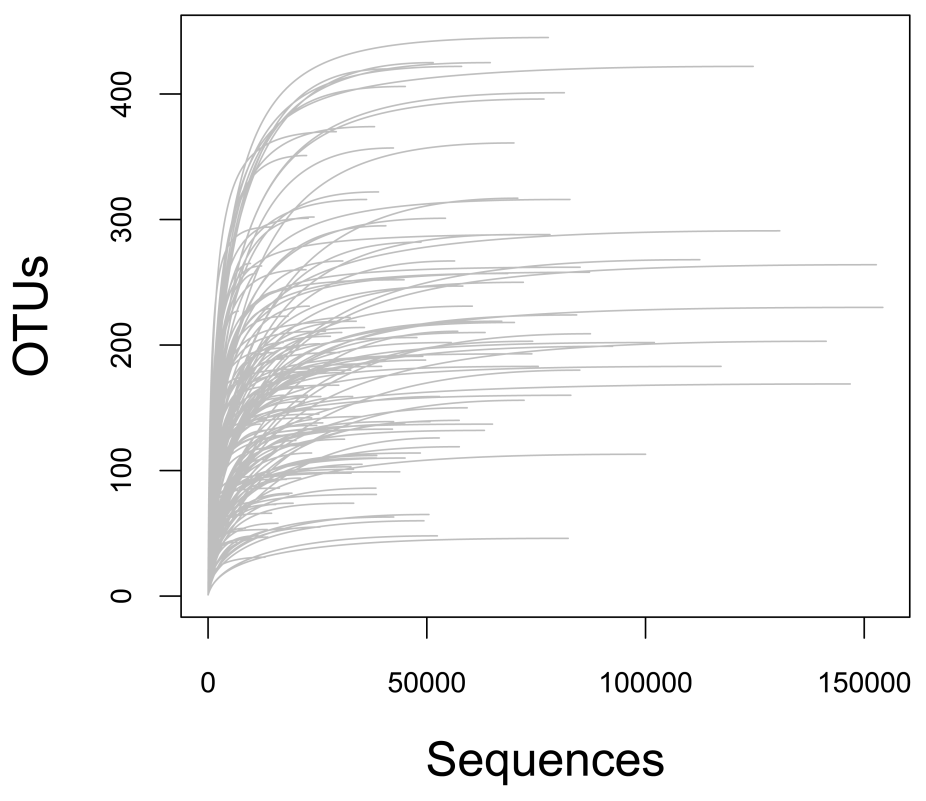


Figure S3. PCoA ordination visualized the influence of subgenera explaining the structure of FEF assemblages. Ellipses indicate the location and dispersion in ordination space for each subgenera (confidence level = 0.90), point presents to each *Ficus* individual.


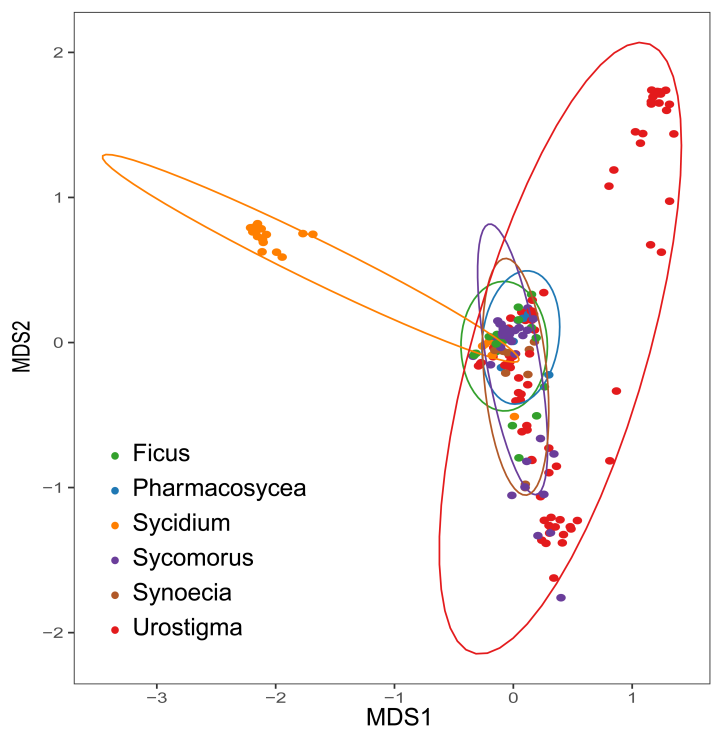


Tree S1: Bayesian tree file in newick format used in this study. Sequences of three loci (*ITS*, *ETS*, and *G3pdh*) were downloaded from NCBI and the model of GTR+I+G was chosen according to AIC.

((Antiaropsis_decipiens:0.09081456634008449,Castilla_elastica:0.09081456634008449):0.03221744531613897,((((((((((((Ficus_abelii:0.005518280211368561,Ficus_stenophylla:0.005518280211368561):0.003911056914076884,Ficus_pandurata:0.009429337125445445):0.0071339691845898855,Ficus_pumila:0.01656330631003533):0.0054279095951844,Ficus_ischnopoda:0.02199121590521973):0.004815109066099669,Ficus_sarmentosa:0.0268063249713194):0.0027657854316926918,((Ficus_esquiroliana:0.013589679731987003,Ficus_laevis:0.013589679731987003):0.00895283245584589,Ficus_langkokensis:0.022542512187832892):0.007029598215179199):0.0015532582815193999,(((Ficus_cyrtophylla:0.012219851329597445,Ficus_subincisa:0.012219851329597445):0.0051166595528261225,(Ficus_subulata:0.011305039069816664,Ficus_tinctoria:0.011305039069816664):0.006031471812606904):0.009019156010594605,(Ficus_henryi:0.010581645824567724,Ficus_tsiangii:0.010581645824567724):0.01577402106845045):0.004769701791513318):0.0032260546344863167,(Ficus_nervosa:0.025991793953084378,Ficus_vasculosa:0.025991793953084378):0.00835962936593343):0.0062189975522627255,(((Ficus_concinna:0.011959056635427522,(Ficus_geniculata:0.005406960894472354,Ficus_virens:0.005406960894472354):0.006552095740955168):0.0026894994386543487,Ficus_superba:0.014648556074081871):0.001817752110319681,Ficus_religiosa:0.016466308184401552):0.02410411268687898):0.001679073415200176,((((((Ficus_auriculata:0.009721223727166998,Ficus_oligodon:0.009721223727166998):0.0019418998376970632,Ficus_beipeiensis:0.011663123564864061):0.004571061121615904,Ficus_sur:0.016234184686479965):0.005144085821817985,Ficus_tikoua:0.02137827050829795):0.001678541472149988,(Ficus_fistulosa:0.017098476852636105,Ficus_prostrata:0.017098476852636105):0.005958335127811833):0.013769781835896451,Ficus_racemosa:0.036826593816344386):0.00542290047013632):0.008563036764016305,(Ficus_hispida:0.0030964187464638413,Ficus_squamosa:0.0030964187464638413):0.04771611230403317):0.006098833086864833,((((((Ficus_altissima:0.01119057764512729,Ficus_elastica:0.01119057764512729):0.004085221017008601,((Ficus_curtipes:0.007350203026193374,Ficus_maclellandii:0.007350203026193374):0.004519825756004475,(Ficus_drupacea:0.010628143174334649,(Ficus_microcarpa:0.0033450888537336753,Ficus_stricta:0.0033450888537336753):0.0072830543206009735):0.0012418856078632006):0.003405769879938042):8.477670501109043E-4,Ficus_benjamina:0.016123565712246796):0.007925532202181205,(Ficus_pisocarpa:0.011607531574093709,Ficus_rumphii:0.011607531574093709):0.012441566340334291):0.0027885615148301145,(Ficus_annulata:0.012402927021353281,Ficus_glaberrima:0.012402927021353281):0.014434732407904834):0.012471974308095397,(Ficus_cyathistipula:0.019680270435158794,Ficus_natalensis:0.019680270435158794):0.019629363302194718):0.017601730400008335):0.06612064751886161);
